# Body-mass index and risk of advanced chronic kidney disease: Prospective analyses from a primary care cohort of 1.4 million adults in England

**DOI:** 10.1371/journal.pone.0173515

**Published:** 2017-03-08

**Authors:** William G. Herrington, Margaret Smith, Clare Bankhead, Kunihiro Matsushita, Sarah Stevens, Tim Holt, F. D. Richard Hobbs, Josef Coresh, Mark Woodward

**Affiliations:** 1 Nuffield Department of Population Health (NDPH), University of Oxford, Oxford, United Kingdom; 2 Oxford Kidney Unit, Churchill Hospital, Oxford University Hospitals NHS Foundation Trust, Oxford, United Kingdom; 3 Nuffield Department of Primary Care Health Sciences (NDPCHS), University of Oxford, Oxford, United Kingdom; 4 Department of Epidemiology, John Hopkins University, Baltimore, MD, United States of America; 5 The George Institute for Global Health, University of Oxford, Oxford, United Kingdom; 6 The George Institute for Global Health, University of Sydney, Sydney, Australia; Istituto Di Ricerche Farmacologiche Mario Negri, ITALY

## Abstract

**Background:**

It is uncertain whether being overweight, but not obese, is associated with advanced chronic kidney disease (CKD) and how the size and shape of associations between body-mass index (BMI) and advanced CKD differs among different types of people.

**Methods:**

We used Clinical Practice Research Datalink records (2000–2014) with linkage to English secondary care and mortality data to identify a prospective cohort with at least one BMI measure. Cox models adjusted for age, sex, smoking and social deprivation and subgroup analyses by diabetes, hypertension and prior cardiovascular disease assessed relationships between BMI and CKD stages 4–5 and end-stage renal disease (ESRD).

**Findings:**

1,405,016 adults aged 20–79 with mean BMI 27.4kg/m^2^ (SD 5.6) were followed for 7.5 years. Compared to a BMI of 20 to <25kg/m^2^, higher BMI was associated with a progressively increased risk of CKD stages 4–5 (hazard ratio 1.34, 95% CI 1.30–1.38 for BMI 25 to <30kg/m^2^; 1.94, 1.87–2.01 for BMI 30 to <35kg/m^2^; and 3.10, 2.95–3.25 for BMI ≥35kg/m^2^). The association between BMI and ESRD was shallower and reversed at low BMI. Current smoking, prior diabetes, hypertension or cardiovascular disease all increased risk of CKD, but the relative strength and shape of BMI-CKD associations, which were generally log-linear above a BMI of 25kg/m^2^, were similar among those with and without these risk factors. There was direct evidence that being overweight was associated with increased risk of CKD stages 4–5 in these subgroups. Assuming causality, since 2000 an estimated 39% (36–42%) of advanced CKD in women and 26% (22–30%) in men aged 40–79 resulted from being overweight or obese.

**Conclusions:**

This study provides direct evidence that being overweight increases risk of advanced CKD, that being obese substantially increases such risk, and that this remains true for those with and without diabetes, hypertension or cardiovascular disease. Strategies to reduce weight among those who are overweight, as well as those who are obese may reduce CKD risk, with each unit reduction in BMI yielding similar relative reductions in risk.

## Introduction

Globally, average body-mass index (BMI) has increased by 0.5kg/m^2^ each decade since 1980.[1] In England, BMI has increased by nearly 1kg/m^2^ in the last decade, such that in 2013 about two-thirds of men and over one-half of women were overweight (ie, had a BMI ≥25kg/m^2^), and about one-quarter of both men and women were obese (ie, had a BMI ≥30kg/m^2^).[2] Chronic kidney disease (CKD) may result from a raised BMI,[3–6] so quantifying by how much BMI affects such risk is important, particularly as CKD is common[7,8] and associated with a wide range of health risks[9–11] and substantial health resource utilisation.[12–14]

Despite its importance, kidney disease is rarely attributed as an Underlying Cause of Death in high-income countries, so cohorts from such populations with fatal follow-up have been unable to study BMI-CKD associations in detail.[15,16] Linkage of two large cohorts to dialysis/transplant registers has enabled examination of the association between BMI and end-stage renal disease (ESRD),[3–6] but these studies were established in the 1960-1990s before treatments which slow CKD progression were commonly used,[17–19] and had limited power to assess how the size and shape of BMI-CKD associations may vary across the full range of BMI in different types of people. A more recent (2004–2006) study of 3.3M United States (US) veterans reported “U”-shaped associations between BMI and risk of “rapid decline in kidney function”[20] challenging results from the other large studies[3–6] by suggesting the optimal BMI to avoid serious kidney failure was in the overweight (BMI 25 to <30kg/m^2^) rather than the lean/“normal” range (BMI 20 to <25kg/m^2^).

The large Clinical Practice Research Datalink (CPRD) dataset of English primary care records includes sufficiently complete measurements of BMI for epidemiological use.[21,22] As English primary care physicians were contracted to screen and maintain registers for CKD,[23] CPRD provides an opportunity to investigate comprehensively current uncertainties about the relevance of BMI to both pre-ESRD advanced CKD and ESRD.[24] Employing strategies recently used by the Global BMI Mortality Collaboration[25] to minimize distortion to associations by conditions which cause weight loss prior to baseline, we aimed to determine the apparent optimum BMI range in relation to the risk of advanced CKD, to test the recent hypothesis that age modifies BMI-kidney disease associations,[20] and to assess whether associations differ among people with and without diabetes, prior cardiovascular disease or uncontrolled hypertension.[6]

## Materials and methods

### Study design and participants

CPRD is an anonymised database of electronic healthcare records from United Kingdom primary care practices using the Vision IT system (about 680 practices, covering all regions and about 8% of the population).[24] CPRD has ethical approvals for general use of these data and the project protocol was approved by the CPRD Independent Scientific Advisory Committee (ISAC); protocol number 15_029R. CPRD data are largely recorded using Read codes which describe lifestyle, symptoms, diagnoses, prescriptions, clinical measurements, medical test results and other treatments. Primary care data from the three-quarters of the English CPRD practices which have linkage to social deprivation indices, Hospitalisation Episode Statistics and national mortality registers form the basis of this cohort. Only CPRD data designated as “research quality” data was included in the study (i.e. data fulfilled CPRD’s “up to standard” and “acceptability” measures.[26])

### Exposure

Adults aged 20–79 with a valid BMI record on or after 1.1.2000 (the date of their first BMI record referred to as the index date for any particular individual) were included provided they had at least one year of data prior to their index date and at least three-years of follow-up thereafter. The first recorded BMI fulfilling all age and date restrictions was used. The index date for each patient was the date of this BMI measurement.

All records of BMI (kg/m^2^), weight (kg) and height (m) were extracted. BMI (weight divided by height squared) (kg/m^2^) was preferentially calculated from the recorded weight (kg) and the nearest recorded adult height (m), or otherwise taken directly from the recorded BMI. Those with a weight, height or BMI (weight divided by height squared) outside, respectively, the range 20-300Kg, 1.3–2.5m or 15-60kg/m^2^ were excluded. Baseline BMI was categorised into five ordinal groups based on World Health Organization (WHO) categories[16]: BMI 15 to <20kg/m^2^, 20 to <25kg/m^2^ (lean); 25 to <30kg/m^2^ (overweight); 30 to <35kg/m^2^ (moderate obesity); and 35 to <60kg/m^2^ (severe and very severe obesity combined).

Some patients may have been weighed because they reported changes in weight, but previous work has shown CPRD BMI records are up to 77% complete, suggesting patients are often routinely weighed (e.g., at registration).[21] Moreover, BMI measurements at the midpoint of follow-up available in 32% (454,078/1,405,016) of this cohort were highly concordant within groups divided by baseline BMI. The overall regression-dilution ratio was 0.87 and was similar across different age subgroups, and among men, women, and those with and without diabetes, prior cardiovascular disease or hypertension. This level of serial correlation is high and similar to that observed in cohorts of apparently healthy adults, indicating that weight changes which may bias associations were uncommon in this CPRD cohort, and that adjustment for regression-dilution bias was unnecessary.[15,27] All analyses are therefore based on a single baseline measure of BMI. Representativeness of the cohort with respect to BMI was confirmed by comparing prevalences of overweight, obese and very severely obese with those reported by Health Survey for England.[2,21]

### Covariates

Covariates were defined from the information recorded before the index date. Smoking categories were current, never, former or unknown. Social deprivation was measured by the Index of Multiple Deprivation (an aggregate of national data on income, employment, education, housing and environment[28]). Prior diabetes was defined by diabetes diagnostic or treatment-related codes, a prescription of anti-diabetic medication or %HbA1c≥6.5 (48mmol/mol). Prior cardiovascular disease included the twelve validated cardiovascular endpoints defined for CPRD by the CArdiovascular research using LInked Bespoke studies and Electronic health Records (CALIBER) programme, including heart failure, coronary artery, cerebrovascular and peripheral arterial diseases.[29] Uncontrolled hypertension was defined as any systolic blood pressure reading of ≥140 mmHg in the year prior to the index date.

### Outcomes

Follow-up was continued from the index date until the earliest of the date of death (77,185 deaths occurred during follow-up, 5.5% of the cohort), date of a record of leaving the practice or 30.3.2014. Incident identified CKD stages 4–5 were derived using internationally accepted clinical definitions[30] and an algorithm incorporating death certificates, inpatient diagnostic or procedural codes, and primary care diagnostic/laboratory test results. Validation work using directly measured glomerular filtration rates (GFR) has suggested creatinine based CKD Epidemiology Collaboration (CKD-EPI) estimated GFR (eGFR) formulae[31] are reliable across a wide range of BMIs,[31,32] so where laboratory results were available, eGFR was calculated from creatinine results. CKD stages 4–5 was accepted if there were at least two eGFR measurements <30mL/min/1.73m^2^, spaced by at least 90 days, with no eGFR result ≥30mL/min/1.73m^2^ in the intervening period. The secondary outcome, incident ESRD, comprised those who died with mention of ESRD, or underwent kidney transplantation or maintenance dialysis (which was distinguished from acute dialysis by a record of CKD stage 5, permanent arteriovenous dialysis access or peritoneal dialysis). Indirect validation using UK-renal registry data confirmed that this ESRD outcome was reliable.

### Statistical analysis

To control for confounding by disease and reverse causality, the first 3 years of follow-up were excluded. To assess the aetiological relevance of baseline BMI to CKD among those without the CKD outcome at baseline, Cox models were fitted with adjustment for relevant confounders (baseline age [continuous], sex, current smoking versus not [which included missing smoking status], and fifths of Index of Multiple Deprivation in England). Subgroup analyses by these confounders were then performed (by baseline age groups 20–39; 40–59; and 60–79 years; sex, current smoking versus not), and social deprivation above versus below the national median). Subgroup analyses then assessed how adjustment for pre-existing mediators of CKD risk (i.e., intermediate factors on the causal pathway) may modify associations. These included diabetes mellitus, uncontrolled hypertension, and prior cardiovascular disease at baseline. Likelihood ratio tests of the interaction were used to assess heterogeneity of associations between subgroups.

Adjusted hazard ratios for each subgroup were then plotted against the mean BMI in each BMI category (e.g., 18.7kg/m^2^ for the 15 to <20kg/m^2^ category) accompanied by a 95% confidence interval (CI) derived using floating absolute risks which allow for direct statistical comparisons to be made between any two groups.[33] Finally, the percentage of cases attributable to being overweight or obese (i.e., the population attributable fraction) was estimated overall, and separately for sex, with adjustment for age and other covariates using the Stata ‘punafcc’ command after Cox regression.[34]

Sensitivity analyses included: increasing the cohort start date to 1.1.2005 (after which BMI measurements were most complete[21]); analyses restricted to known non-smokers [25] and exclusion of the first 7 years of follow-up (to further control for distorting effects of weight changes before baseline); applying a stricter definition of CKD stages 4–5 (which required the latest available eGFR result to be <30mL/min/1.73m^2^); adjustment for pre-existing mediators of risk; and using Fine and Gray models to account for the competing risk of non-ESRD mortality. Consistency with the Cox proportional hazards assumption was checked graphically using log(–log) survival curves. All statistical analyses used Stata v14 (StataCorp 2015) or R v3.2.2 (www.R-project.org).

## Results

Of the 4,078,746 adults aged 20–79 years included in CPRD between 2000 and 2011 with linked data, 2,080,994 had a measure of BMI. After excluding 645,044 with less than 3 years follow-up and 30,934 with extreme BMI measures or missing data on social deprivation, 1,405,016 remained (S1 Fig). Mean age was 49 (SD 16) years and 58% (817,720/1,405,016) were women (Table 1). Overall, 71% of men (415,480/587,296) and 57% of women (464,895/817,720) had a BMI ≥25 kg/m^2^, including 27% of men (160,377/587,296) and 26% of women (214,505/817,720) who were obese (Table 1).

**Table 1:** Baseline characteristics and follow-up time of the cohort overall and by category of body-mass index.

			Baseline body-mass index (kg/m^2^)			
	≥15,<20	≥20,<25	≥25,<30	≥30,<35	≥35,<60	All
**Number of patients**	74,875	449,766	505,493	245,729	129,153	**1,405,016**
**Mean body-mass index, kg/m**^**2**^	18.7 (1.0)	22.8 (1.4)	27.3 (1.4)	32.1 (1.4)	39.4 (4.2)	**27.4 (5.6)**
**Confounders**						
Mean baseline age, years	41.4 (17.2)	46.5 (16.4)	51.0 (15.2)	50.6 (14.6)	47.7 (14.2)	**48.7 (15.8)**
Female sex	57,540 (77%)	295,285 (66%)	250,390 (49%)	129,644 (53%)	84,861 (66%)	**817,720 (58**%**)**
Cigarette smoking						
Current	20,139 (27%)	95,725 (21%)	88,434 (17%)	38,625 (16%)	18,234 (14%)	**261,740 (19**%**)**
Former	8842 (11%)	77,716 (17%)	113,312 (22%)	55,315 (23%)	23,804 (18%)	**287,589 (20**%**)**
Never	21,912 (29%)	156,821 (35%)	164,821 (33%)	71,236 (29%)	33,740 (26%)	**448,530 (32**%**)**
Missing	24,382 (33%)	119,504 (27%)	138,926 (27%)	80,553 (33%)	53,375 (41%)	**416,740 (30%)**
Higher than average social deprivation	33,771 (45%)	179,039 (40%)	208,343 (41%)	112,979 (46%)	67,305 (52%)	**601,437 (43**%**)**
**Pre-existing mediators of risk**						
Diabetes mellitus	1746 (2.3%)	17,945 (4.0%)	37,598 (7.4%)	26,643 (11%)	18,453 (14%)	**102,385 (7.3**%**)**
Uncontrolled hypertension	9978 (17%)	99,430 (26%)	175,881 (40%)	101,703 (49%)	55,021 (53%)	**442,013 (37**%**)**
Prior cardiovascular disease	4187 (5.6%)	33,881 (7.5%)	58,026 (11%)	30,038 (12%)	13,475 (10%)	**139,607 (10**%**)**
Chronic kidney disease outcomes at baseline or within the first 3 years of follow-up*						
CKD stage 4 or 5	292 (0.4%)	1,901 (0.4%)	2,800 (0.6%)	1,546 (0.6%)	904 (0.7%)	7,443 (0.5%)
End-stage renal disease	136 (0.2%)	720 (0.2%)	790 (0.2%)	410 (0.2%)	205 (0.2%)	2,261 (0.2%)
**Median follow-up, years**	7.0 (4.8–9.7)	7.4 (5.1–10.1)	7.6 (5.3–10.2)	7.6 (5.3–10.3)	7.7 (5.2–10.4)	**7.5 (5.2–10.2)**
**Deaths**	4,814 (6.4%)	21,697 (4.8%)	28,602 (5.7%)	14,539 (5.9%)	7,533 (5.8%)	**77,185 (5.5%)**

CKD = Chronic kidney disease. Data are number (%) or mean (standard deviation) or median (interquartile cutoffs).

* Patients with these outcomes were excluded from the main analyses for that outcome.

The prevalence of a BMI ≥25kg/m^2^ increased from age 20, peaking in men at ages 50–54 at 76% (50,417/66,210) and in women at ages 65–69 at 67% (37,335/55,581; S2 Fig). The age-adjusted prevalences of prior diabetes for those with a BMI of 20 to <25kg/m^2^ versus 30 to <35kg/m^2^ were 6% versus 12% for men, and 3% versus 9% for women. Higher prevalences of uncontrolled hypertension, and cardiovascular disease were also evident among those with higher BMI (S1 Table).

Median follow-up was 7.5 years (interquartile interval 5.2–10.2). After exclusion of the first 3 years of follow-up, 11,490 (0.82%) participants were identified as developing CKD stages 4–5, among whom 1687 (0.12%) started maintenance renal replacement therapy or died with ESRD mentioned. CKD stages 4–5 were identified much more commonly among older adults, with incidence rising steeply from the age of about 60. At each age, men were at higher risk of CKD stages 4–5 (S3 Fig) and ESRD (S4 Fig) than women.

In prospective analyses adjusting for confounders, compared to those with a BMI of 20 to <25kg/m^2^, those with BMIs of 25 to <30kg/m^2^, 30 to <35kg/m^2^ and ≥35kg/m^2^ had 34%, 94%, and 210% higher risk of developing CKD stages 4–5, respectively (corresponding hazard ratios: 1.34, 95% confidence interval 1.30–1.38; 1.94, 1.87–2.01; and 3.10, 2.95–3.25). Adjustment for potential effect mediators (diabetes mellitus, uncontrolled hypertension, and prior cardiovascular disease) reduced these hazards by about 30–40%, but clear positive associations remained (1.20, 1.16–1.23; 1.54, 1.48–1.60; and 2.19, 2.08–2.31 respectively; Table 2). Sensitivity analyses restricting to never-smokers, left-censoring by 7 years, only including the period when BMI measures were more complete, using stricter definitions of CKD stages 4–5, and adjusting for competing risk of non-ESRD mortality had no substantial effect on associations (S2 Table), and justified our initial conclusions.

**Table 2:** Association between body-mass index and the risk of advanced chronic kidney disease by different levels of adjustment.

		Crude model		Adjusted for age and sex		Adjusted for confounders		Adjusted for other mediators of risk	
Baseline BMI (kg/m^2^)	Number of outcomes*	HR	(95% CI)	HR	(95% CI)	HR	(95% CI)	HR	(95% CI)
**Chronic kidney disease stages 4 or 5**									
**≥15,<20**	**256**	0.72	(0.64–0.81)	0.95	(0.84–1.07)	**0.92**	**(0.81–1.04)**	0.98	(0.86–1.12)
**≥20,<25**	**2305**	1.00	(0.96–1.04)	1.00	(0.96–1.04)	**1.00**	**(0.96–1.04)**	1.00	(0.96–1.04)
**≥25,<30**	**4457**	1.68	(1.63–1.73)	1.34	(1.30–1.38)	**1.34**	**(1.30–1.38)**	1.20	(1.16–1.23)
**≥30,<35**	**2795**	2.15	(2.07–2.23)	1.97	(1.89–2.04)	**1.94**	**(1.87–2.01)**	1.54	(1.48–1.60)
**≥35,<60**	**1677**	2.43	(2.32–2.55)	3.18	(3.03–3.34)	**3.10**	**(2.95–3.25)**	2.19	(2.08–2.31)
**End-stage renal disease**									
**≥15,<20**	**60**	1.02	(0.79–1.32)	1.35	(1.05–1.74)	**1.32**	**(1.02–1.70)**	1.33	(0.99–1.78)
**≥20,<25**	**380**	1.00	(0.90–1.11)	1.00	(0.90–1.11)	**1.00**	**(0.90–1.11)**	1.00	(0.90–1.11)
**≥25,<30**	**639**	1.45	(1.35–1.57)	1.14	(1.06–1.24)	**1.14**	**(1.05–1.23)**	1.00	(0.93–1.09)
**≥30,<35**	**357**	1.66	(1.50–1.84)	1.39	(1.26–1.55)	**1.36**	**(1.23–1.51)**	1.02	(0.92–1.14)
**≥35,<60**	**251**	2.20	(1.94–2.49)	2.32	(2.05–2.63)	**2.22**	**(1.96–2.52)**	1.44	(1.26–1.64)

BMI = Body-mass index. HR = hazard ratio. CI = confidence interval.

* Excludes outcomes occurring at baseline or within the first 3 years of follow-up. Confounders included: baseline age (continuous), sex, current smoking and fifths of social deprivation; pre-existing other mediators of risk included: diabetes, uncontrolled hypertension and prior cardiovascular disease at baseline. CIs were estimated using the method of floating absolute risk that allows appropriate statistical comparisons to be made between any two groups.

Associations between BMI and ESRD were also generally positive but were on average weaker than for the CKD stages 4–5 outcome. Compared to those with a BMI of 20 to <25kg/m^2^, those with BMIs of 25 to <30kg/m^2^, 30 to <35kg/m^2^ and ≥35kg/m^2^ were associated with an 14%, 36%, and 122% increased risk of ESRD respectively (hazard ratios: 1.14, 1.05–1.23; 1.36, 1.23–1.51; and 2.22, 1.96–2.52; Table 2). Furthermore, there was evidence of an increased risk in those with a BMI <20kg/m^2^ which remained apparent even when the first 7 years of follow-up were excluded (S2 Table).

**Fig 1:**
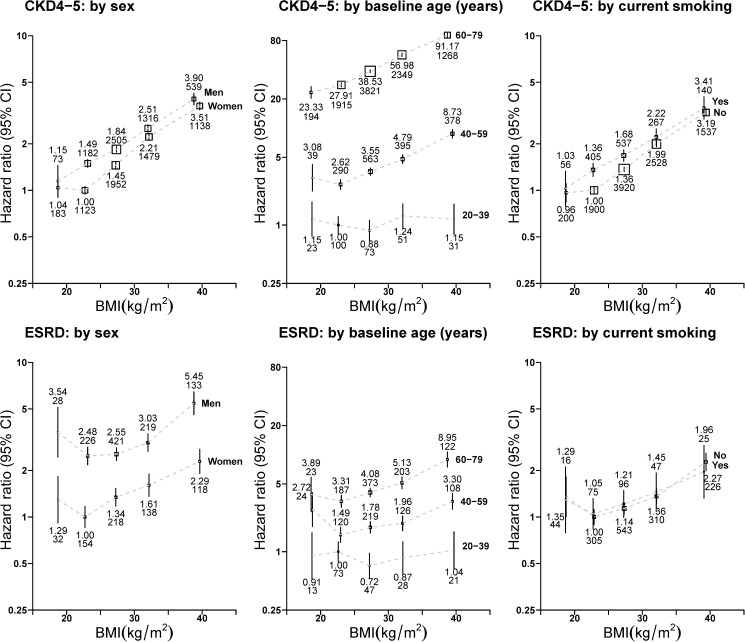
Association between baseline body-mass index and risk of advanced chronic kidney disease by sex, age and smoking status at baseline. BMI = body-mass index. CKD4-5 = CKD stage 4 or 5. ESRD = end-stage renal disease. CI = confidence interval. Analyses excluded those with outcomes at baseline or during the first 3 years of follow-up. Hazard ratios were all adjusted or stratified by age (continuous), sex, current smoking and level of deprivation (by fifths) and are plotted against the mean BMI in each BMI category. Boxes are plotted so that the area is proportional to the inverse variance of the floated hazard ratio. Error bars indicate 95% CIs and are accompanied by the hazard ratio (upper number) and number of outcomes (lower number).

Men, current smokers and those from areas with above national average levels of social deprivation were more likely to develop CKD stages 4–5 when compared to women, non-smokers and those from below average levels of social deprivation. There were generally positive log-linear associations between BMI and CKD stages 4–5 in the overweight and obese BMI range which were similar in both shape and relative strength when analyses were stratified by smoking status and level of social deprivation. However, for age, the BMI association was relatively flat at ages 20–39 and became steeper with older age (interaction test by age p<0.0001). There was also statistical evidence that the slope was slightly steeper for women than men (interaction test by sex p<0.0001; Fig 1), although the difference is size and shape of the associations between sexes was small. Similar differences in risk between levels of covariates were also apparent for the ESRD outcome although there were insufficient outcomes to detect statistically significant interactions (Fig 1).

**Fig 2:**
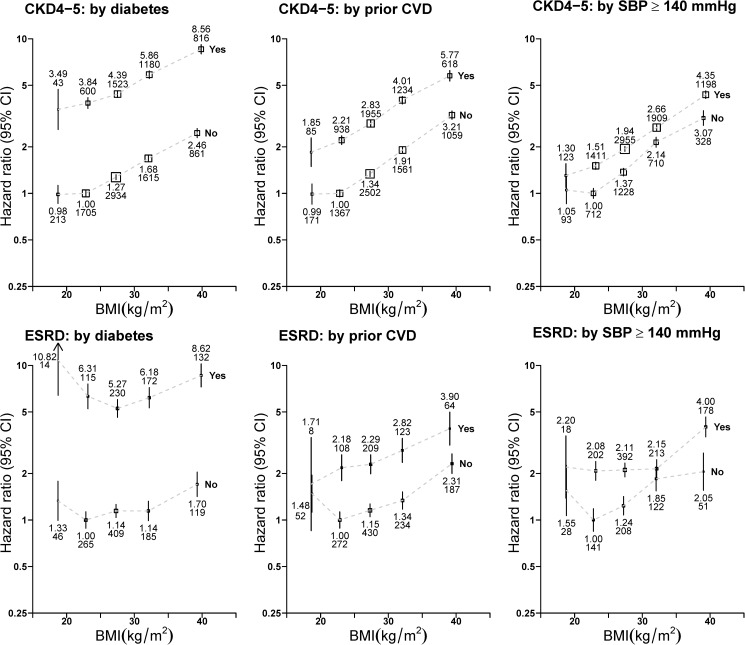
Association between baseline body-mass index and risk of advanced chronic kidney disease by diabetes, prior cardiovascular disease and uncontrolled hypertension at baseline. BMI = body-mass index. CKD4-5 = CKD stage 4 or 5. ESRD = end-stage renal disease. CVD = cardiovascular disease. SBP = systolic blood pressure. CI = confidence interval. Analyses excluded those with outcomes at baseline or during the first 3 years of follow−up. SBP analyses exclude 218,466 participants with a missing measurement. Hazard ratios were all adjusted or stratified by age (continuous), sex, current smoking and level of deprivation (by fifths) and are plotted against the mean BMI in each BMI category. Boxes are plotted so that the area is proportional to the inverse variance of the floated hazard ratio. Error bars indicate 95% CIs and are accompanied by the hazard ratio (upper number) and number of outcomes (lower number).

Fig 2 presents BMI associations by relevant potential effect mediators and demonstrates that those with a baseline history of diabetes or prior cardiovascular disease were, respectively, about 3.5-times and 2.1-times more likely to develop CKD stages 4–5 during follow-up than those without. However, approximately parallel association lines demonstrate that the shape and relative strength of associations between higher BMI and CKD stages 4–5 were independent of these CKD risk factors. The shape and relative strength of associations between baseline BMI and ESRD also appeared to be unmodified when analyses were stratified by potential mediators of risk.

Table 3 presents the fraction of advanced CKD risk attributable to being overweight or obese in this primary care cohort for those aged 40–79 (little association existed between BMI and risk of CKD below this age). Estimates suggest that 33% (95% CI 30–35%) of new CKD stages 4–5 in 2000–2014 was attributable directly or indirectly to a BMI ≥25kg/m^2^ (falling to 24% when adjusted for potential effect mediators). Since the estimated hazard ratios were bigger in women, this proportion was slightly higher in women (39%, 36–42%) compared to men (26%, 22–30%). Weaker associations between BMI and ESRD meant smaller population attributable fractions for this outcome, but a BMI of ≥25kg/m^2^ still accounted for 20% (13–27%) of ESRD (Table 3).

**Table 3:** Percentage of advanced chronic kidney disease attributable to being overweight or obese at ages 40–79 in English primary care 2000–2014.

	Number of cases	Prevalence of BMI ≥25kg/m^2^ among cases	Hazard ratio (95% CI): BMI ≥25 vs. <25kg/m2		Population attributable fraction (95% CI)	
**Chronic kidney disease stage 4–5**						
Men	5495	78%	1.50	(1.41–1.60)	26%	(22–30%)
Women	5717	79%	2.00	(1.88–2.13)	39%	(36–42%)
**Overall**					**33%**	**(30–35%)**
**End-stage renal disease**						
Men	939	78%	1.28	(1.10–1.50)	17%	(7–26%)
Women	566	75%	1.52	(1.25–1.84)	25%	(14–35%)
**Overall**					**20%**	**(13–27%)**

BMI = body-mass index. CI = confidence interval. In this cohort, prevalence of overweight, obesity and very severe obesity in men and women were similar to levels observed in the recent representative Health Survey for England,[2,21] and the age structure between 40–79 years closely mirrored recent national Census data.[35] The population attributable fraction was calculated using the Stata punafcc function after Cox regression and adjustment for confounders (i.e., age, cigarette smoking and level of social deprivation).

## Discussion

This large modern English cohort of 1.4 million primary care registered adults with a recent BMI measurement has provided an opportunity to study BMI and CKD with more precision than has previously been possible. With higher BMI, the risk of developing advanced CKD rose steeply and progressively. Compared to lean weight adults, those who were overweight, but not obese, were at about 40% increased risk, those with moderate obesity were at doubled risk, and those with severe obesity were at 3-times the risk of developing advanced CKD. Above a BMI of 25kg/m^2^, associations were generally log-linear and similar in shape among people with and without other key CKD risk factors, including diabetes, hypertension and cardiovascular disease. There was also direct evidence that being overweight increases the risk of advanced CKD overall and separately among people with these comorbidities.

We used linked primary care, secondary care, and mortality data to provide reliable “real-world” identification of CKD outcomes (S3 and S4 Figs). The identification of pre-ESRD advanced CKD outcomes and excluding acute kidney injury was particularly important for this research question as these earlier CKD stages may be less likely to be affected by the altered appetite and weight loss that can result from ESRD and its causes. This, and other strategies to minimize distorting effects from unmeasured disease which may cause both weight loss before baseline and affect risk, may explain why this study has shown the optimum BMI to minimize CKD risk in the lean range. This result is consistent with reports from older cohorts which controlled for such biases with very long follow-up,[3,6] but directly challenges findings from a recent study of 3.3M US Veterans that being overweight is protective against important changes in kidney function.[20] The US Veterans study used an outcome called “rapid decline in kidney function” which was defined as an eGFR slope of >5mL/min/1.73m^2^. This outcome occurred in 8% of participants of the US Veterans cohort,[20] which is an order of magnitude higher than the prevalence of advanced CKD (defined as a persistent eGFR <30mL/min/1.73m^2^) both in the US,[7] and in this CPRD cohort. The outcome of “rapid decline in kidney function” may therefore include a large proportion of kidney disease which is attributable to acute kidney injury/temporary fluctuations in creatinine, and may not be a reliable surrogate for progressive CKD. Nevertheless, like the US Veterans cohort, we also found that BMI associations with our kidney disease outcomes become steeper with increasing age.[20] This result may simply reflect longer BMI exposure in older adults, but as albuminuria measurements were available in only a small proportion of the CRPD cohort, it was not possible to assess whether younger kidneys were truly not affected by increased BMI using this early marker of kidney disease.

Observational associations may not represent cause and effect; however, BMI-CKD associations are biologically plausible and there is some randomized evidence to support causal assertions. Trials and Mendelian randomization experiments have both confirmed that obesity is a cause of type 2 diabetes [36–39] and therefore, by extrapolation, diabetic kidney disease.[40] Additionally, the Look Action for Health in Diabetes (Look AHEAD) trial showed that intensive lifestyle intervention reduced weight by 8% (on average 4Kg) and reduced the risk of developing “very-high-risk CKD” by about 30% in people who were already obese and had type 2 diabetes.[41] Our results also show that the long-term detrimental effects of high BMI on kidneys were similar in those with or without diabetes, supporting the hypothesis that a raised BMI is also a key risk factor for non-diabetic CKD.[42] In its severest forms obesity can be a primary renal diagnosis.[43] A key mechanism for such obesity-associated glomerulopathy is intraglomerular hypertension.[44–47] The consequent mechanical stress results in glomerular hypertrophy[48] with podocyte death and focal segmental glomerulosclerosis.[49–51] It has also been proposed that pre-diabetes levels of blood glucose may be a metabolic podocyte stressor,[48,52] that adipose tissue-derived leptin is directly toxic to renal tubules,[53] and that increased renal sinus fat can compress major renal vessels.[54–56] However, the precise mechanisms by which increased BMI appears to be causing CKD need to be better understood in order for appropriate intervention to be developed.

We found strong positive BMI-CKD associations which are consistent qualitatively with results from much older cohorts with very long follow-up (25 years).[3–6] Quantitatively, however, the relative risks were somewhat smaller. These more modest BMI associations in CPRD (2000–2014) do not appear to be a consequence of increased variability in BMI (resulting from measurement error or weight change), as the correlation between baseline and follow-up BMI within BMI groupings was as high as observed in large studies of apparently healthy adults.[15] It is possible that relative risks derived from CPRD underestimate true hazards by virtue of the comparatively shorter follow-up time (7 years), but smaller relative risks could also result from recent improvements in the opportunity for detection[23] and treatment of diabetes,[17] hypertension and albuminuria.[18,19]

Despite its large size and the methods employed to minimize bias, there are study limitations. First, the main outcome required an indication for diagnostic blood testing. It is therefore possible that some participants with CKD were not excluded at baseline or not identified during follow-up. Reassuringly however, indications for CKD screening in the UK did not include being overweight/obese,[23] and the relative strengths of BMI-CKD associations were no different between those with and without screening indications (which included diabetes, hypertension, and cardiovascular disease).[23] This suggests any differential outcome ascertainment was unlikely. Secondly, during the course of this study nationwide re-calibration of laboratory creatinine assays to be traceable by isotope dilution mass spectrometry was performed. Among those with CKD stages 4–5, the older assays tended to overestimate eGFR by about 1-2mL/min/1.73m^2^.[57] Therefore CKD incidences in this study may slightly underestimate true incidence. Importantly, however, assay changes should not have affected relative risks. Lastly, other primary care studies have found relatively more women have a BMI measurement than men[58] and such selection bias may explain our study’s relatively low proportion of men compared to the English population (particularly below the age of 40 years). The consistent shape of BMI associations with CKD risk across subgroups and the robustness of results in sensitivity analyses suggest the presented relative risks are generalizable and reliable. However, residual bias resulting from the cohort being selected on being weighed during a healthcare visit cannot be excluded.

In this cohort, prevalence of overweight, obesity and very severe obesity in men and women were all similar to levels observed in the recent nationally-representative Health Survey for England,[2,21] and the age structure between 40–79 years closely mirrored recent national Census data.[35] We therefore used these CPRD data to estimate that, in the years 2000–2014 in England, about two-fifths of advanced CKD in women and one-quarter in men aged 40–79 may be attributable directly or indirectly to being overweight or obese. Such a large proportion strengthens the rationale for national efforts to implement effective lifestyle-modification programs[38,39] and emerging[59] novel fiscal strategies to reduce calorie intake.[60] Importantly, the log-linear associations observed above 25kg/m^2^ suggest that any unit reduction in BMI that can be achieved may reduce relative risks of advanced CKD by a similar amount among those who are overweight, as well as those who are obese, irrespective of the presence of diabetes, hypertension or cardiovascular disease.

## Supporting information

S1 Table Age-standardised prevalence of baseline prior disease by body-mass index and sex. Data are all %. *prevalences directly standardised to the 5-year age structure of the overall study population
(TIF)

S2 Table Association between baseline body-mass index with risk of advanced chronic kidney disease outcomes (sensitivity analyses). BMI = Body-mass index. Unless stated, analyses excluded those with the outcome at baseline or during the first 3 years of follow-up. Hazard ratios (HR) are adjusted for confounders including baseline age (continuous), sex, current smoking, and fifths of social deprivation. Confidence intervals (CI) are group-specific CIs that allow appropriate statistical comparisons to be made between any two groups.
(TIF)

S1 Fig Study population selection. CPRD = Clinical Practice Research Datalink; HES = Health Episode Statistics; ONS = Office of National Statistics; BMI = body-mass index.
(TIF)

S2 Fig Prevalence of overweight and obesity by age and sex. BMI = body-mass index. Symbols are for men (circles) and women (triangles). 95% confidence intervals (CI) are smaller than the plotted symbols.
(TIF)

S3 Fig Age−specific incidence rates of chronic kidney disease stages 4 or 5 by age and sex. BMI = body-mass index. Symbols are for men (circles) and women (triangles). Vertical lines represent 95% confidence intervals, but are often smaller than the symbols. Age−specific incidence rates are plotted at the mean age of outcome in each age group. For incidence rates the youngest age bands are combined into 10−year bands (20−29 and 30−39 years) because of low numbers of events; the oldest age band is 85+ years.
(TIF)

S4 Fig Age−specific incidence of end-stage renal disease in the study population (left panel) and of treated end-stage renal disease in the UK in 2009 (right panel) by age and sex. Symbols for men (circles) and women (triangles). Age−specific incidence rates in the study population and 95% confidence intervals (vertical lines) are plotted at the mean age of outcome in each age group (left panel). The youngest age bands were combined because of low event numbers at ages 20−29 and 30−39 years. UK rates per million population in 2009 (right panel) taken from Chapter 1 of the UK Renal Registry's 13th Annual Report.
(TIF)
