# Supplementary figures and images for: Body-mass index and risk of advanced chronic kidney disease: Prospective analyses from a primary care cohort of 1.4 million adults in England
Source: PLoS One. 2017 Mar 8;12(3):e0173515. doi: 10.1371/journal.pone.0173515 (PMC5342319; doi:10.1371/journal.pone.0173515)

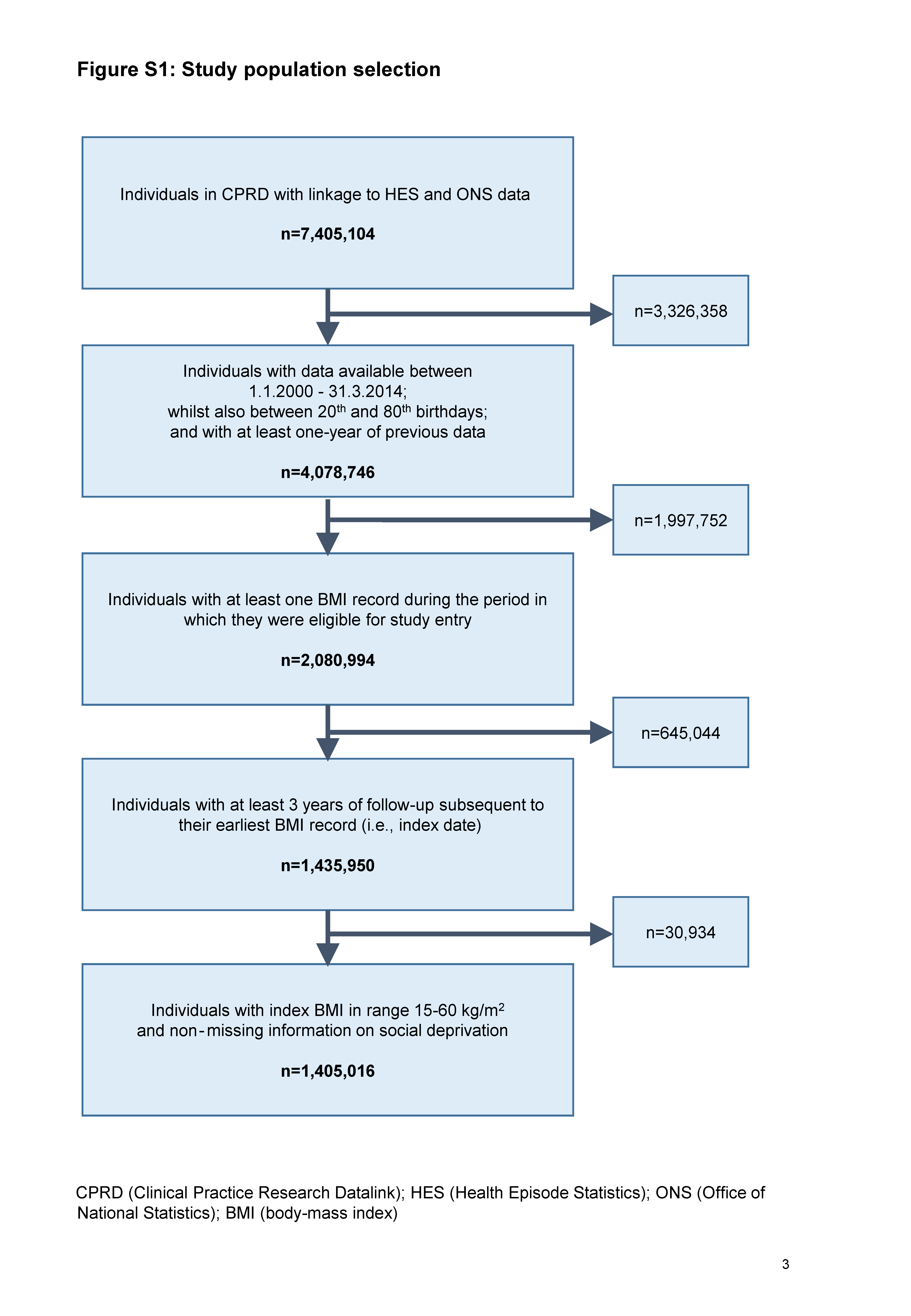

Supplement: S1 Fig — CPRD = Clinical Practice Research Datalink; HES = Health Episode Statistics; ONS = Office of National Statistics; BMI = body-mass index. (TIF) [file pone.0173515.s003.tif]
